# A systematic review of outcomes assessed in randomized controlled trials of surgical interventions for carpal tunnel syndrome using the International Classification of Functioning, Disability and Health (ICF) as a reference tool

**DOI:** 10.1186/1471-2474-7-96

**Published:** 2006-12-05

**Authors:** Christina Jerosch-Herold, José C de Carvalho Leite, Fujian Song

**Affiliations:** 1 School of Allied Health Professions, University of East Anglia, Norwich, UK

## Abstract

**Background:**

A wide range of outcomes have been assessed in trials of interventions for carpal tunnel syndrome (CTS), however there appears to be little consensus on what constitutes the most relevant outcomes. The purpose of this systematic review was to identify the outcomes assessed in randomized clinical trials of surgical interventions for CTS and to compare these to the concepts contained in the International Classification of Functioning, Disability and Health (ICF).

**Methods:**

The bibliographic databases Medline, AMED and CINAHL were searched for randomized controlled trials of surgical treatment for CTS. The outcomes assessed in these trials were identified, classified and linked to the different domains of the ICF.

**Results:**

Twenty-eight studies were retrieved which met the inclusion criteria. The most frequently assessed outcomes were self-reported symptom resolution, grip or pinch strength and return to work. The majority of outcome measures employed assessed impairment of body function and body structure and a small number of studies used measures of activity and participation.

**Conclusion:**

The ICF provides a useful framework for identifying the concepts contained in outcome measures employed to date in trials of surgical intervention for CTS and may help in the selection of the most appropriate domains to be assessed, especially where studies are designed to capture the impact of the intervention at individual and societal level. Comparison of results from different studies and meta-analysis would be facilitated through the use of a core set of standardised outcome measures which cross all domains of the ICF. Further work on developing consensus on such a core set is needed.

## Background

Carpal tunnel syndrome is the most common peripheral entrapment neuropathy and a frequent cause of disability in the upper extremity[1]. Surgical release or decompression rates in the USA amount to 400,000 to 500,000 per year [2] and 43 to 74 per 100,000 in the UK[3]. Conservative treatment of CTS also accounts for a large proportion of resources and includes splinting, nerve gliding, ultrasound and carpal bone mobilisation[2]. Whilst the reported success rates of carpal tunnel release range from 70 to 90 percent there is little agreement on how results should be evaluated[4].

The evidence from trials evaluating the outcomes from surgical and non-surgical interventions is extensive[2,5], however the pooling of results as required for meta-analysis has been impeded by the wide range of outcome measures used. Outcomes from carpal tunnel release have been assessed in many different ways including objective nerve conduction studies, clinical measures of sensibility, muscle function and dexterity, patient-reported symptoms and perceived function and the impact on daily activity, work and leisure. It has also been noted, that there is a lack of consensus among clinicians over what constitutes the most reliable, valid and responsive instruments to evaluate outcomes in carpal tunnel syndrome[4,6].

Outcome measures should not only capture the impact of the disorder on body structure (impairments) but also on activities and participation as defined in the International Classification of Functioning, Disability and Health (ICF)[7]. The ICF is part of the family of International Classifications developed by the World Health Organisation[7]. It provides a framework which uses unifying terminology for the classification of diseases and their effect on body structure and functioning, activities and participation. The ICF complements existing classifications systems such as the International Statistical Classification of Diseases and related health problems – ICD-10. The ICF is particularly useful to understand and measure health outcomes which look beyond mortality and morbidity [7] as it reflects a biopsychosocial perspective describing the impact of disease from an individual and societal perspective. Its applications in health are manifold, including outcome evaluation and work is underway linking the measurement of health status in different patient populations with the ICF[8].

Given the wide range of outcomes that have been employed in trials of surgical interventions for CTS and the lack of consensus among clinicians on what are considered the most relevant outcomes to assess [4]it was hypothesised that linking the outcomes to the ICF would highlight where outcome measures overlap in the concepts and domains which they assess and whether the existing outcomes assessed encompass all aspects of functioning as conceptualised by the ICF. The results of such a review may inform the selection of outcome domains and respective instruments for future trials on interventions in CTS. Similar work has already been undertaken in the fields of back pain, osteoarthritis [9] and stroke [10] and this in turn has resulted in the development of consensus on core sets for outcome assessment in these populations.

## Methods

A systematic review was performed to identify the outcomes assessed and concepts contained in the measures used in trials of interventions for CTS, and to relate these concepts to the ICF as a reference tool.

### Search strategy

The bibliographic databases *Medline *(January 1966 to July 2005), *CINAHL *(January 1982 to July 2005), *AMED *(January 1985 to July 2005) were searched using the following MeSH terms: randomized controlled trial, controlled clinical trial, carpal tunnel decompression, carpal tunnel release, carpal tunnel surgery. The titles and abstracts of articles retrieved through the search were checked applying the eligibility criteria as defined below. In order to crosscheck the sensitivity of the search strategy and identify any studies not retrieved through the search the bibliographies of a Cochrane review on surgical interventions for CTS were also examined[5].

### Study selection criteria and procedures

The review considered all published studies if they met the following eligibility criteria: randomized or quasi-randomized trials, the interventions were surgical, the patients had a diagnosis of CTS made through clinical symptoms with or without confirmatory electrodiagnostic testing, and the outcomes assessed were described. Studies designed solely for the purpose of evaluating the effectiveness of different local anaesthesia on pain during or within 48 hours of surgery were excluded.

English language publications only were included and regardless of the time of the publication. The purpose of this review was not to assess the methodological quality of these trials as this has been done previously[5,11,12], but to identify the outcome domains assessed and the instruments or scales employed for this purpose, irrespective of whether these were standardised or not. Outcome measures extraction was carried out using a standard form to obtain data on the following aspects: study design, experimental and control interventions, length of follow-up, sample size, the outcome domains assessed pre- and post-operatively and respective instruments or scales employed. Each article was independently read by two reviewers and a data extraction form completed. Any discrepancies between reviewers were discussed and agreed.

## Results

137 studies were identified through the initial search. After checking the abstracts and further review of the full-text article a total of 28 studies which met inclusion criteria were identified [13-40] A summary of the main study characteristics is shown in Table 1.

### Review of studies and outcome domains assessed

The studies, conducted between 1985 and 2005, were all designed to evaluate the relative effectiveness of different surgical techniques such as endoscopic carpal tunnel release (ECTR) or open carpal tunnel release with or without epineurotomy. Standard open carpal tunnel release (OCTR) was the control intervention in 27 of the 28 trials. A total of 2558 hands in 2232 patients were studied in 28 studies. The length of follow-up after decompression varied greatly between the studies, ranging from 4 weeks to 2 years. The mean follow-up time was 37 weeks with 13 studies reporting follow-up at 1 year or longer. There was no apparent association between the type of outcomes assessed and other study characteristics such as the type of intervention studied, the length of follow-up or the country of the study.

A wide range of outcomes were reported and these were classified according to the three ICF domains: i) measures of impairment of body function and body structure; ii) measurers of activity limitations and iii) measures of participation restriction (see Tables 2 and 3). The most commonly assessed outcome domain was symptom resolution (27 studies) using either non-standardised methods (19 studies) or the standardised and disease-specific instrument, the Symptom Severity Scale of the Boston Carpal Tunnel Questionnaire (BCTQ)[41]. The second most commonly assessed outcomes were complications (including scar adhesions, pillar pain and wound infection) and motor function, measured either as composite power grip (19 studies) and/or pinch grip (14 studies) with dynamometry and/or using manual muscle testing (MMT) and/or presence or absence of wasting of the thenar muscles. Sensibility was assessed in 15 studies and included touch threshold with monofilaments or the pressure specifying device (PSSD), two-point discrimination and vibration. Other outcomes within the impairment domain included range of movement of wrist or fingers, nerve conduction studies and interstitial pressures of the carpal canal.

At the level of activity limitations, hand function was assessed with timed dexterity tests in three studies or through self-reported use of hand in activities of daily living including the Functional Status Score of the BCTQ (12 studies). Measures of participation used in the trials were return to work which featured as a primary or secondary outcome measure in 15 studies and satisfaction (7 studies).

The level of reporting on the actual methods of assessment varied between studies. In several studies standardised outcome measures were used but with none or minimal detail on the actual instrument used, method of administration or reference to literature on standardised protocols. For example, 11 studies assessed two-point discrimination with only 4 studies stating the instrument used and one study describing the method (Table 4).

The BCTQ, a standardised patient-based outcome measure for use in CTS, started to feature only in studies published in 2000 and later, despite the original paper by Levine dating back to 1993. Non-standardised methods of assessing symptom resolution continued to be reported in 6 studies post 2000 and included assessing a range of symptoms which patients were asked to rate on a 3 or 4 point ordinal scale or to indicate as present or absent.

For some domains there were inconsistencies in the methods of quantifying the outcome. Return to work was a primary or secondary outcome in 15 studies and was often reported as the number of the days from surgery to resumption of employment. However, this is likely to vary between studies and depends on factors such as type of work undertaken (manual versus non-manual) employment status (self-employed versus employee) and variations in healthcare systems and how 'sick notes' are issued. The time taken to return to work also does not indicate whether someone is able to resume the activities without pain or discomfort and to the satisfaction of the individual and/or his employer. Furthermore, work as a measure of participation is not relevant to those patients who are not in employment or retired.

### Linking outcomes assessed in CTS trials with the ICF

Current understanding of the pathophysiology of CTS together with the clinical manifestation of signs and symptoms allows the specific body structures and functions to be identified which are implicated in this disease. Depending on the severity of their symptoms, patients are also affected in their ability to carry out activities (activity limitations) and participation in work or leisure (participation restrictions). These in turn are also influenced by personal and environmental factors [7].

**Figure 1:**
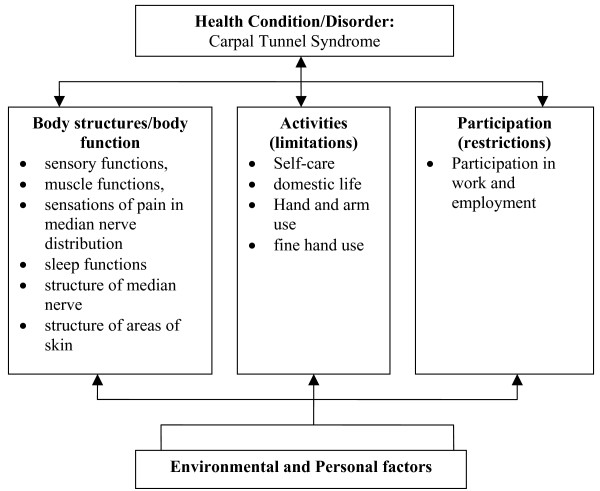
Interaction of concepts of the ICF and relation to outcome domains assessed in CTS trials (adapted from ICF, WHO 2002).

**Figure 2:**
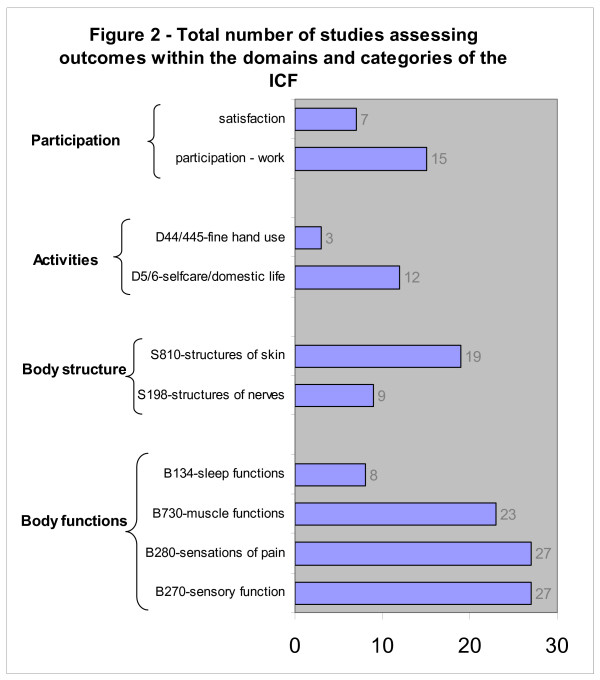
Total number of studies assessing outcome within the domains and categories of the ICF.

Using the ICF as a framework, firstly the codes and categories within health and health-related domains relevant to CTS were identified (see figure 1). Secondly the outcomes assessed in the 28 studies retrieved were mapped to the relevant domains and categories of the ICF. Where instruments contained subscales and several items these were individually assigned to the relevant category (see table 5). The frequency with which the domains and categories of the ICF were assessed in all 28 studies is presented in Figure 2.

The outcomes assessed in the 28 studies together covered all three domains of the ICF, that is, impairments of body structure and function, activity limitations and participation restrictions. However the majority of outcomes assessed focused on impairments of body structure and function, that is, sensory functions, sensations of pain, motor functions and sleep functions. Activity limitations and participation restrictions were assessed far less frequently in trials and included the patient-rated Functional Status Scale of the BCTQ, timed hand dexterity tests and self-reported time taken to resume activities of daily living, mostly self-care and household chores. Number of days taken to return to work or satisfaction were the only measures of participation restrictions.

## Discussion

This systematic review has identified a wide range of outcome domains which were assessed in trials of surgical interventions and using a variety of methods and instruments. Based on the total count of studies which assessed different domains, self-reported resolution of symptoms, grip strength and return to work were the most frequently assessed outcomes.

The focus of these trials on assessing outcomes at the level of body function and structure and lesser use of measures of activity and participation concurs with findings from Gummerson et al [42] who reviewed trials of upper extremity disorders published in 4 journals over 11 years and totalling 92 studies. Only 41% of those studies reported outcomes on activity and participation.

Aspects such as the impact of CTS on functioning and health-related quality of life was included in only a few trials and limited to measures of days taken to return to work. A number of region-specific, patient-based outcome instruments which include questions about appearance, use of hand in self-care, work and leisure have been developed in the last 10 years. Examples of these are the Patient Evaluation Measure (PEM)[43] and the Disabilities of the Arm, Shoulder and Hand Questionnaire (DASH)[44]. They are not disease-specific measures but region-specific and their validity has been tested in patient populations with a range of upper extremity musculoskeletal disorders, including CTS. Questions such as those from the DASH optional work module '*did you have difficulty using your usual technique at work?' *or '*did you have difficulty in doing your work as well as you would like?' *evaluate participation in work and provide useful additional information beyond the simple quantification of sick days. There are also a number of generic measures of health-related quality of life, such as the SF-36 [45] which capture participation and the psychosocial impact of the disease or disorder and which can be used in CTS patients. Of the RCTs reviewed, none used generic measures of participation or other health-related quality of life or health status measures.

Whilst it is important to obtain a comprehensive assessment of the impact of CTS on body function and structure as well as activity and participation, the use of a large battery of instruments also increases the burden on the patient and the tester. There was some overlap between the concepts assessed in trial outcomes. For example, in the domains body functioning, the functions of muscles were assessed by manual muscle testing of the abductor pollicis brevis muscle, dynamometry for pinch and power grip strength and degree of thenar wasting. In several trials two or more of these measures were used concurrently. A review of the evidence is needed on which of these methods and instruments is most valid, reliable and responsive which, in turn, reduces assessment burden and redundancy of similar outcome measures.

The use of the disease-specific measure, the BCTQ is becoming more established in recent trials. A systematic review of the psychometric properties of the BCTQ indicated that it is valid for the population, has good reliability and is responsive [46] and should replace any other non-standardised methods for assessing self-reported symptom resolution and functional status. The two subscales together encompass the two domains of the ICF – body function and activities. The use of clinician-derived measures of motor and sensory function was high yet the lack of detail on instruments, methods of testing and reference to published protocols or literature on psychometric properties of these tests raises questions over whether these were employed in the same way, thus hindering comparability of results. There are several advantages to using standardised outcome measures: if data about the reproducibility and population-specific validity of an instrument are known the use of it as a primary outcome measure is justified; knowledge of the responsiveness of an outcome measures can inform sample size calculations which are important to ensure that studies are adequately powered; and finally, the results from several studies can be compared and pooled if the same outcomes have been assessed using the same standardised methods and instruments.

This review has some limitations: we considered RCTs only in this review, based on the assumption that in well-designed trials careful attention would be paid to the selection of outcome measures. There are however a number of large follow-up and cohort studies which also report outcomes from surgical decompression and inclusion of these studies may have highlighted additional outcome domains and instruments.

## Conclusion

The ICF provides a useful framework for identifying the concepts contained in outcome measures employed to date in trials of surgical intervention for CTS. It can help in the selection of the most appropriate domains to be assessed, especially where studies are designed to capture the impact of the intervention at individual and societal level. The findings of this review on surgical outcomes indicate that studies to date have focused primarily on assessment of impairment and less on the activity limitations and participation restrictions. It is important that consensus is achieved on which outcome measures should be used for which domains and on the standardisation of methods. A minimum set of outcome measures should include patient-reported scales of symptom severity and functional status such as the BCTQ, clinical measures of motor and sensory function and everyday performance in self-care, work and leisure as well as health-related quality of life. Further work is needed to review the psychometric properties of existing instruments in CTS populations and to develop consensus on a core set of outcome measures to be used in future clinical trials for CTS which crosses all three domains of the ICF.

## Competing interests

The author(s) declare that they have no competing interests.

## Authors' contributions

CJH conceived of the original idea, obtained funding and participated in the search, review and drafting of the manuscript. JCCL carried out the searching and reviewing of studies and helped draft the manuscript. FS provided expert advice on systematic reviewing and contributed to the manuscript. All authors read and approved the final manuscript.

## Pre-publication history

The pre-publication history for this paper can be accessed here:



## Figures and Tables

**Table 1:** Summary of randomized controlled trials of surgical interventions for carpal tunnel syndrome

First Author/date	Country	Experimental intervention	Control	Sample size		Length of follow-up (weeks)	blinded assessor
						
				Patients	hands		
Holmgren-Larsson (1985)	Sweden	OCTR with epineurotomy.	OCTR	48	48	36	
Lowry (1988)	USA	OCTR with neurolysis	OCTR	41	50	12	
Mackinnon l (1991)	Canada	OCTR with neurolysis	OCTR	59	64	52	
Agee (1992)	USA	ECTR	OCTR	122	147	26	
Brown (1993)	USA	ECTR	OCTR	145	169	12	
Erdmann (1994)	UK	ECTR	OCTR	71	105	52	
Foulkes (1994)	USA	OCTR with epineurotomy	OCTR	33	36	52	
Sennwald (1995)	Switzerland	ECTR	OCTR	47	47	12	
Dumontier (1995)	France	ECTR	OCTR	96	96	36	
Jacobsen (1996)	Sweden	ECTR	OCTR	29	32	36	
Leinberry (1997)	USA	OCTR with epineurotomy	OCTR	44	50	52	
Citron (1997)	UK	OCTR (L-incision)	OCTR	47	47	52	
Nakamichi (1997)	Japan	OCTR with Ultrasound	OCTR	103	103	104	
Brüser (1999)	Germany	OCTR (short incision.)	OCTR	80	80	6	
Mackenzie (2000)	USA	ECTR	OCTR	26	36	4	
Trumble (2002)	USA	ECTR	OCTR	147	192	52	
Jugovac (2002)	Croatia	OCTR (short incision)	OCTR	72	72	12	
Shum (2002)	USA	OCTR with Flexor tenosyn	OCTR	87	88	52	
Ferdinand (2002)	UK	ECTR	OCTR	25	50	52	
Borisch (2003)	Germany	OCTR with epineurotomy	OCTR	273	307	52	
Macdermid (2003)	Canada	ECTR	OCTR	123	123	52	
Saw (2003)	UK	ECTR	OCTR	123	123	12	
Helm (2003)	UK	'Knifelight' ECTR	OCTR	82	82	6	
Wong (2003)	China	'limited' OCTR	ECTR	30	60	52	
Battacharya (2004)	UK	Knifelight' ECTR	OCTR	26	52	6	
Dias (2004)	UK	Lengthening of flex ret.	OCTR	26	52	25	
Kharwadkar (2005)	UK	OCTR absorbable sutures	OCTR	33	40	12	
Lorgelly (2005)	UK	Minimally invasive OCTR	OCTR	194	208	104	

TOTAL n = 28				2232	2558		

ECTR = endoscopic carpal tunnel release; OCTR = Open carpal tunnel release; flex ret. = flexor retinaculum; tenosyn = tenosynovectomy

**Table 2:** Outcomes assessed in RCTs of surgical interventions for CTS at the level of impairment in body structures and body functions of the ICF

**First Author/date**	**Symptoms**	**Motor function**				**Sensory function**			**Body structure**		
	
	Pain, sensory, motor symptoms, sleep disturbance	Grip	Pinch	MMT/thenar atrophy	RoM	Touch threshold	2PD	Vibration	Nerve conduction or interstitial pressure	Skin incisional complications	Pillar pain, causalgia
Holmgren (1985)	NS										
Lowry (1988)	NS										
Mackinnon (1991)	NS										
Agee (1992)	NS										
Brown (1993)	NS										
Erdmann (1994)	NS										
Foulkes (1994)	NS										
Sennwald (1995)	NS										
Dumontier (1995)	NS										
Jacobsen (1996)	NS										
Leinberry (1997)											
Citron (1997)	NS										
Nakamichi (1997)	NS										
Brüser (1999)	NS										
Mackenzie (2000)	BCTQ										
Trumble (2002)	BCTQ										
Jugovac (2002)	NS										
Shum (2002)	BCTQ										
Ferdinand (2002)	NS										
Borisch (2003)	NS										
Macdermid (2003)	BCTQ										
Saw (2003)	BCTQ										
Helm (2003)	NS										
Wong (2003)	NS										
Battacharya (2004)	NS										
Dias (2004)	BCTQ										
Kharwadkar (2005)	BCTQ										
Lorgelly (2005)	BCTQ										

Total = 28 studies	27	19	14	10	3	9	11	3	9	19	18

NS = non-standardised, BCTQ – Boston Carpal Tunnel Questionnaire; MMT – manual muscle testing; 2PD – two = point discrimination

**Table 3:** Outcomes assessed in RCTs of surgical interventions for CTS at the level of activity limitations and participation restrictions of the ICF

**First Author/date**	**Activities – self-care, household Fine hand use**			**Participation/satisfaction**	
	
	Dexterity (Jebsen, Purdue)	Use of hand in ADL	Functional Status scale BCTQ	Time to return to work	Satisfaction
Holmgren (1985)					
Lowry (1988)					
Mackinnon (1991)					
Agee (1992)					
Brown (1993)					
Erdmann (1994)					
Foulkes (1994)					
Sennwald (1995)					
Dumontier (1995)					
Jacobsen (1996)					
Leinberry (1997)					
Citron (1997)					
Nakamichi (1997)					
Brüser (1999)					
Mackenzie (2000)					
Trumble (2002)					
Jugovac (2002)					
Shum (2002)					
Ferdinand (2002)					
Borisch (2003)					
Macdermid (2003)					
Saw (2003)					
Helm (2003)					
Wong (2003)					
Battacharya (2004)					
Dias (2004)					
Kharwadkar (2005)					
Lorgelly (2005)					

Total = 28 studies	3	5	7	15	7

BCTQ – Boston Carpal Tunnel Questionnaire, ADL – activities of daily living

**Table 4:** Level of reporting of instrument, method of assessment and reference to literature for standardised clinical measures (numbers in bold indicate number of studies, square bracketed numbers refer to study number in reference list),

Outcome categories and instruments		Instrument named/described	Method of administration described	Cited reference on psychometric properties/protocol	Assessed but no details given	Total number of studies
Grip strength	Dynamometer (Jamar, Baseline, NK or B&L) vigorimeter	**15 **[16–20,24,26–28,31,30–35,37–38]	**2 **[19, 38]	**2 **[19,33]	**4 **[15,21,25,36]	**19**
Pinch strength		**11 **[16–20,26–28,31,33,38]	**2 **[19, 38]	**1 **[33]	**3 **[15, 25,36]	**14**
Manual muscle testing of thenar muscles (MRC, AOA scale)		n/a	**6 **[15–17, 25, 28, 31]	**3 **[15, 23, 28]	**2 **[29, 32]	**10**
Range of motion	goniometry	**1 **[38]	**2 **[21, 31]	**1 **[31]	**0**	**3**
Sensibility	Touch threshold (SWMT or PSSD)	**9 **[15–17, 19, 25,26, 28, 30, 33]	**1 **[19]	**4 **[15,17,19,33]	**0**	**9**
	2 – point discrimination	**4 **[15, 17, 19, 31]	**1 **[19]	**4 **[15, 17, 19, 31]	**7 **[14, 22, 23, 25, 28, 30, 36]	**11**
	vibration	**3 **[15,19]	**1 **[19]	**2 **[13, 15]	**0**	**3**
Hand dexterity (Jebsen-Taylor test, Purdue and 9-hole peg test)		**3 **[28,31,38]	**0**	**2 **[28, 38]	**0**	**3**
Pain Questionnaires (McGill)		**1 **[33]	n/a	**1 **[33]	**0**	**1**
Symptom Severity Scale (BCTQ		**8 **[27, 28,30, 33, 34, 38–40]	n/a	**8 **[27, 28,30, 33, 34, 38–40]	**0**	**8**
Functional Status Scale (BCTQ)		**7 **[27, 28,30, 34, 38–40]	n/a	**7 **[27, 28,30, 34, 38–40]	**0**	

MRC – Medical Research Council, AOA – American Orthopedic Association, SWMT – Semmes-Weinstein Monofilament Test, PSSD – pressure-specifying device; BCTQ – Boston Carpal Tunnel Questionnaire; n/a – not applicable

**Table 5:** Classification of outcomes assessed in randomized controlled trials of surgical interventions for CTS according to the domains and categories of the ICF

**ICF – codes and category titles**	**Outcome measures – domains and instruments/scales used**
**Body function**	
B270 – sensory function of sensing temperature, vibration, pressure and noxious stimuli	touch thresholds (monofilaments or pressure-specifying device)
	vibration (vibrometry or tuning forks)
	two-point discrimination, static and moving
	Self-reported numbness at night and day (BCTQ-Symptom Severity Scale or non-standardised method)
B280 – Sensations of pain (and paraesthesias)	frequency and intensity of pain (BCTQ-Symptom Severity Scale or non-standardised method)
	Visual Analogue Scale for pain
	McGill Pain Questionnaire
B730 – Muscle functions	Manual muscle testing
	Thenar wasting/atrophy
	Power and pinch grip dynamometry
	self-reported weakness (BCTQ-Symptoms Severity Scale)
B134 – sleep functions, including amount and quality of sleep	Waking from nocturnal symptoms (BCTQ-Symptom Severity Scale)

**Body structures**	
S198-Structures of the nervous system, specified (median nerve)	Nerve conduction studies/electrophysiological recording, interstitial pressures
S810-structure of areas of skin (incisionally related wounding and scarring on anterior heel of hand)	Pillar pain, scar tenderness, scar sensitivity, wound inflammation, appearance

**Activities**	
D5 – self-care D6 – domestic life	Use of hand in self-care activities (BCTQ-Functional Status Scale or non-standardised method)
D440 – fine hand use D445 – hand and arm use	Timed hand function/dexterity tests (Jepsen, Purdue, 9-hole peg)

**Participation**	
Participation in work and employment	Days taken to return to work
Satisfaction	Patient Satisfaction
